# Impact of COVID-19 Restrictions in Portugal: A Questionnaire to Municipal and Animal Association Shelters

**DOI:** 10.3390/ani11092532

**Published:** 2021-08-28

**Authors:** Eduarda Gomes-Neves, Sara Marques, Adélia Alves-Pereira, Pedro Osório, Alexandra Müller, Cláudia S. Baptista

**Affiliations:** 1 Instituto de Ciências Biomédicas Abel Salazar (ICBAS), University of Porto, Rua Jorge Viterbo Ferreira 228, 4050-313 Porto, Portugal; emneves@icbas.up.pt (E.G.-N.); saramarques@cibio.up.pt (S.M.); 2 Centro de Estudos de Ciência Animal (CECA), Instituto de Ciências, Tecnologias e Agroambiente da Universidade do Porto (ICETA), Rua D. Manuel II, Apartado 55142, 4051-401 Porto, Portugal; 3 Centro de Investigação em Biodiversidade e Recursos Genéticos (CIBIO), Laboratório Associado em Biodiversidade e Biologia Evolutiva (InBIO), Universidade do Porto, Campus Agrário de Vairão, Rua Padre Armando Quintas No.7, 4485-661 Vairão, Portugal; 4 Shelter Veterinarian, 4050-313 Porto, Portugal; adeliamedicina.abrigo@gmail.com (A.A.-P.); pedrocunhaosorio@gmail.com (P.O.)

**Keywords:** COVID-19 restrictions, animal shelters, animal welfare, cats, dogs, management, survey, feed supply, public funding

## Abstract

**Simple Summary:**

The purpose of this study was to investigate the impact of COVID-19 restrictions in animal shelters in Portugal, namely, if there were differences between Municipal and Association shelters. A questionnaire was sent to both types of animal shelters. Responses were obtained from all areas of Portugal, and significant differences were reported on the numbers of animals and volunteers, funding and feed supply. Animal welfare aspects, such as abandonment, adoption, exercise and animal interaction, were not significantly different. Municipal shelters seem to be more vulnerable to operational and instruction constraints, whereas Associations were more dependent on volunteer work, funding and, consequently, feed supply. When facing an unpredicted health and social event such as the pandemic, organizations should rely on previously defined preparedness and contingency plans.

**Abstract:**

The COVID-19 pandemic has an indirect impact on the health and welfare of animals. The aim of this work was to investigate the effect of COVID-19 on Municipal and Association animal shelters. A questionnaire was sent to 97 Municipal shelters and 65 Associations. Questions focused on public funding, management and animal welfare during COVID-19 restrictions. The response rate was 43.3% (42/97) for Municipal shelters and 38.5% (25/65) for Associations. Municipal shelters (67%) received over 80% of public funding, whereas 68% of the Associations received less than 50%. During the COVID-19 restrictions, financial difficulties were observed by 52% of Associations and 5% of Municipal shelters, and a lack of volunteers was observed by 56% of Associations and 17% of Municipal shelters. Operational difficulties were indicated by 43% of Associations and 12% of Municipal shelters, and a lack of instructions was observed by 31% of Municipal shelters and 4% of Associations. No significant differences were obtained on abandonment, adoption, clinical support, exercise and interaction. Decreased feed supply was reported by 40% of Associations and 5% of Municipal shelters. During the COVID-19 restrictions, Municipal shelters were more affected by the lack of instructions, and Associations were more affected by external factors such as a decrease in feed supply, volunteers and funding. Preparedness and contingency plans seem essential to face unpredicted crises.

## 1. Introduction

Infection with the SARS-CoV-2 virus (COVID-19) spread all over the world in early 2020. Countries are still struggling to contain this virus, whilst trying to address the resulting public health, social and economic challenges [1]. In Portugal, COVID-19 was officially recognized on the 26 February 2020, and the first cases were confirmed on 2 March. The immediate psychological impact of the pandemic on the human population has been evaluated in Portugal, as well as some socio-economic risk factors identified by an online survey [2]; however, the effects on animals remain to be studied. Challenges to human mental health during a crisis may impair the capability to provide adequate care to domestic animals or to perceive when animal welfare is at risk [3].

In Portugal, since the first emergency state was officially declared on the 19 March 2020, several general restrictions were implemented to human movements, namely, the circulation between 11 p.m. and 5 a.m., or between areas with very high incidence (with or without a sanitary fence). However, these major restrictions were not applicable to health emergencies and veterinary services. Moreover, to the best of our knowledge, there were no specific restrictions to animal shelters in general.

The COVID-19 pandemic had an indirect impact on the health and welfare of almost all animals, including wildlife, livestock, laboratory and companion animals [1,3,4,5,6,7,8]. According to the COVID-19 Thematic Platform on Animal Welfare, the main narratives on companion and shelter animals were the shelter influx of relinquished animals, China banning dog meat, shelter economics and the shortage of adoptable pets [5]. In some countries, people abandoned their animals due to fear of the virus, even despite the lack of evidence that pets can spread SARS-CoV-2 to humans [7,9]. Conversely, an increasing interest in pet adoption, in particular dogs, following the pandemic has also been reported [10].

To respond to animal abandonment and to promote adoption, there are two main types of animal shelters for companion animals in Portugal, registered in the National Veterinary Authority (DGAV) webpage. Municipal shelters were originally designated “municipal kennels” and had a historical role in the rabies and population control of stray dogs. Nowadays, these shelters continue to play a pivotal role in veterinary public health and animal welfare and are still governed by the municipalities and headed by municipal veterinarians. Several aspects of legislation have changed, e.g., the use of euthanasia has been restricted, and Municipal shelters have been renamed Official Centers of Animal Intake. The other type of shelters includes animal protection organizations, which are legally registered as Animal Shelter Associations. These Associations are non-governmental organizations, depending on donations and voluntary work. Many of these shelters also provide veterinary care. Similar to other countries, both shelter types are committed to animal health and welfare, promoting the control of stray animal populations as well as responsible ownership [11,12].

The aim of this work was to investigate the effect of COVID-19 restrictions on funding, management and companion animal welfare in Municipal animal shelters and Associations. We hypothesized that COVID-19 restrictions had a different impact on both shelter types.

## 2. Materials and Methods

### 2.1. Questionnaire

To characterize animal shelters on a national level, a questionnaire was developed by all authors in weekly online meetings starting in late 2019. In early 2020, the questions reached consensus and were considered finalized. Taking into consideration the impending COVID-19 pandemic, a further 10 questions on the impact of COVID-19 restrictions on animal shelters were added. The Ethics Committee of ICBAS-CHUP approved the questionnaire in October 2020. The target population of the questionnaire were all animal shelters in Portugal. As there is no national registry of shelters, we used two approaches to create a list of existing animal shelters: (a) the Municipal shelters were approached by asking the Association of Municipalities to disseminate the questionnaire to their associates, and (b) a publicly available list of Animal Associations that are registered by the National Veterinary Authority (DGAV) until the end of 2019 was used. The existence of each listed Animal Association was checked by searching websites and social network platforms (e.g., Facebook). All Animal Associations sheltering companion animals were included, whereas breeders and Associations keeping hunting dogs were excluded. After this process, a list with the email addresses of 97 Municipal Animal shelters and 65 Animal Associations was obtained.

The questionnaire was delivered using an online platform, and the link sent to Animal shelters [13]. The survey was open from the 1 February until the 31 March 2021, with an email reminder being sent on the 15 March. Respondents were kept anonymous, and the confidentiality of individual responses was guaranteed. The questions were applicable to the whole period in which COVID-19 restrictions were enforced, from the 19 March 2020 to the 31 March 2021.

#### 2.1.1. Characterization of Animal Shelters

The first set of questions referred to the characteristics of animal shelters, which are constant and unrelated to the COVID-19 pandemic. These included questions on the type of animal shelter (Municipal shelter, Association), geographical location by region (12 specified regions, no response), the number of animals (dogs, cats), the proportion of funding from public sources (>80%, 50–80%, <50%, no response) and the number of volunteers working at the shelter (none, 1–4, 5–10, >10). Only one option could be selected. Shelters with volunteers were additionally questioned if the availability of volunteers was regular (yes, no, not applicable). All the responses referred to the moment the questionnaire was completed.

#### 2.1.2. Impact of COVID-19 Restrictions

The second set contained 10 questions on the impact of COVID-19 restrictions on animal shelters, and respondents were asked to select those which applied to their context. Selected items were classified automatically as “yes”, and non-selected items as “no”. The option “no response” was available to allow for a distinction between a true “no” and a “non-response”. The questionnaire is available as Supplementary Materials.

### 2.2. Data Analysis

The proportions of the responses of Animal Associations and of Municipal shelters were displayed graphically. To identify any relationship between the outcome variables (public funding, categories of numbers of volunteers, proportion of selected items of the 10 COVID19-related questions) due to the predictor (being a Municipal shelter or an Association), the N-1 Chi-squared test was run in the online calculator Medcalc [14,15,16]. Medcalc allows one to compare proportions of two samples. For example, the item “Over 80% of public funding” was selected by 66.7% of Municipal shelters (n = 42 respondents) and by 0% of Associations (n = 25 Associations). The values of % and number of respondents (n) of Municipal shelters represented Sample 1 and those of Associations represented Sample 2. As only two proportions were compared, all chi-square analyses have 1 degree of freedom (d.f.). Numbers of cats and dogs were compared between shelter types using the independent-samples Mann–Whitney U test in SPSS version 26.0 [17].

## 3. Results

### 3.1. Response Rate and Geographical Distribution

**Figure 1:**
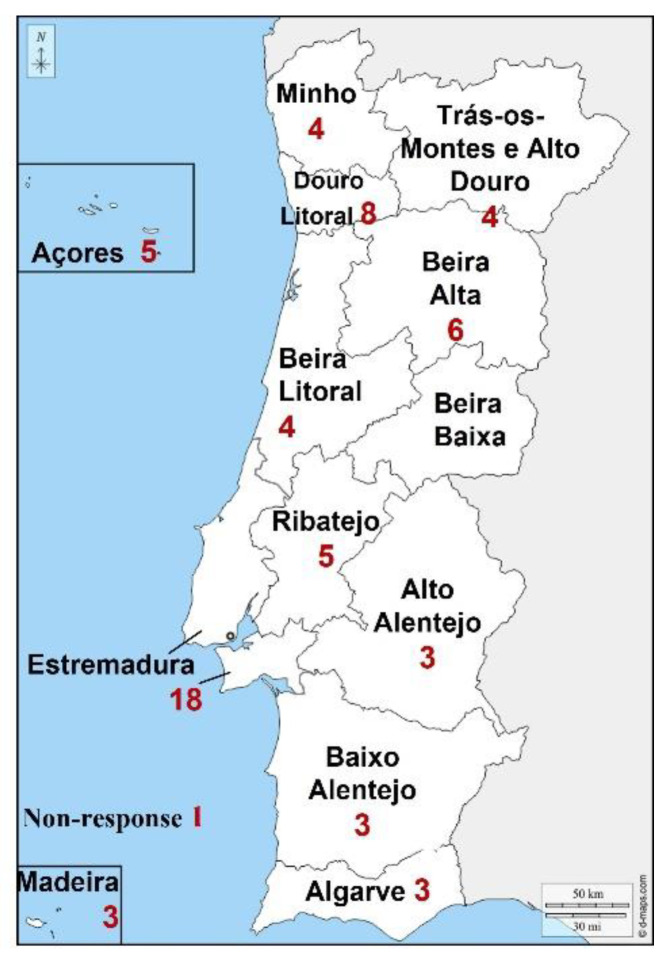
Geographical distribution of completed questionnaires (n = 67) directed to Animal shelters in Portugal in March/April 2021 (Map adapted from https://d-maps.com/carte.php?num_car=24972&lang=pt (accessed on 26 July 2021)).

A total of 67 animal shelters, 42 Municipal shelters and 25 Associations, completed the questionnaire. The response rate was 43.3% (42/97) by Municipal shelters and 38.5% (25/65) by Associations (d.f. 1, *p* = 0.5). Individuals responsible for animal shelters from all regions responded to this survey (Figure 1).

### 3.2. Characterization of Animal Shelters

**Figure 2:**
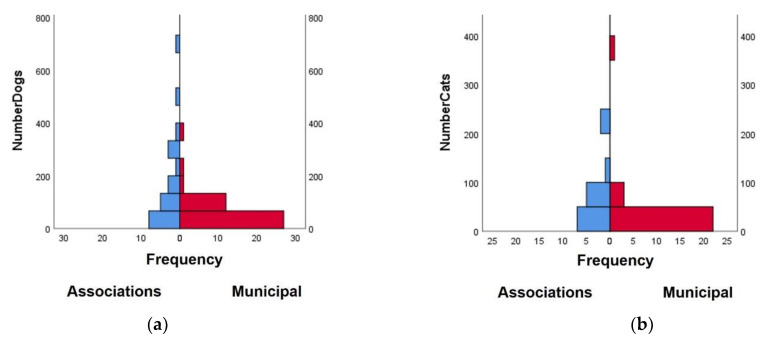
Number of (**a**) dogs and (**b**) cats housed in Association and Municipal animal shelters, as measured by the frequency of questionnaire responses. Data gathered by a questionnaire targeted to Municipal and Non-Municipal shelters (Associations) in March/April 2021.

The median number of animals was significantly higher in Associations than in Municipal shelters (Figure 2). The median number of dogs in Associations was 225 compared to 60 in Municipal shelters (*U* = 271.5; *p* = 0.04). The median number of cats was 48 in Associations and 11 in Municipal shelters (*U* = 86; *p* = 0.03).

**Figure 3:**
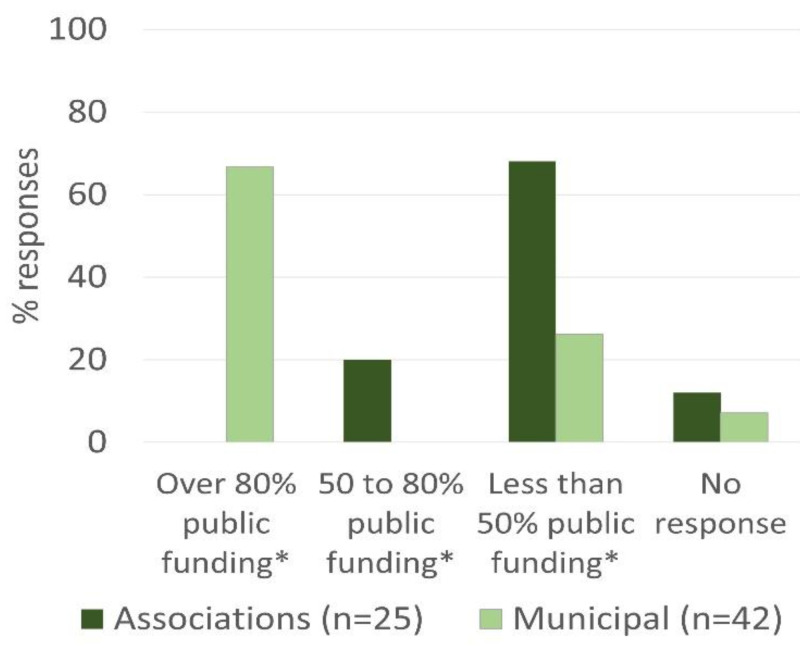
Public funding available to animal shelters. Data gathered by a questionnaire targeted to Municipal and Non-Municipal shelters (Associations) in March/April 2021. * Indicates statistically significant difference between Municipal shelters and Animal Associations (d.f. 1, *p* < 0.05).

The public funding of animal shelters is shown in Figure 3. Over 80% of public funding was indicated by 66.7% of Municipal shelters and by 0% of Associations (d.f. 1, *p* < 0.0001). Between 50 and 80% of public funding was indicated by 20% of the Associations and by 0% of Municipal shelters (d.f. 1, *p* = 0.0028). Less than 50% of public funding was indicated by 26.2% of Municipal shelters and by 68% of Associations (d.f. 1, *p* = 0.0009). Three (12%) Associations and three (7%) Municipal shelters did not respond to this item (d.f. 1, *p* = 0.5).

**Figure 4:**
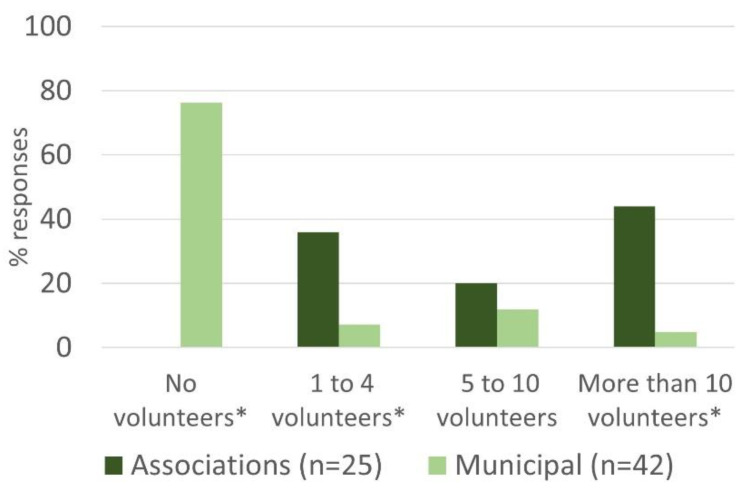
Number of volunteers working at animal shelters. Data gathered by a questionnaire targeted to Municipal and Non-Municipal shelters (Associations) in March/April 2021. * Indicates statistically significant difference between Municipal shelters and Animal Associations (d.f. 1, *p* < 0.05).

The number of volunteers working at animal shelters is illustrated in Figure 4. The option “No volunteers” was only selected by Municipal shelters (76.2%, d.f. 1, *p* < 0.0001). Between 1 and 4 volunteers were indicated by 36% of Associations and by 7.2% of Municipal shelters (d.f. 1, *p* = 0.0032). Between 5 and 10 volunteers were indicated by 20% of Associations and by 11.9% of Municipal shelters (d.f. 1, *p* = 0.37). Over 10 volunteers were indicated by 44% of Associations and by 4.8% of Municipal shelters (d.f. 1, *p* = 0.0001).

Considering the availability of volunteers, 16 (64%) Associations and 9 (21.4%) Municipal shelters indicated a regular offer of volunteers (d.f. 1, *p* = 0.0005). A total of 20 (47.6%) Municipal shelters selected the option “Not applicable”.

### 3.3. Impact of COVID-19 Restrictions

**Figure 5:**
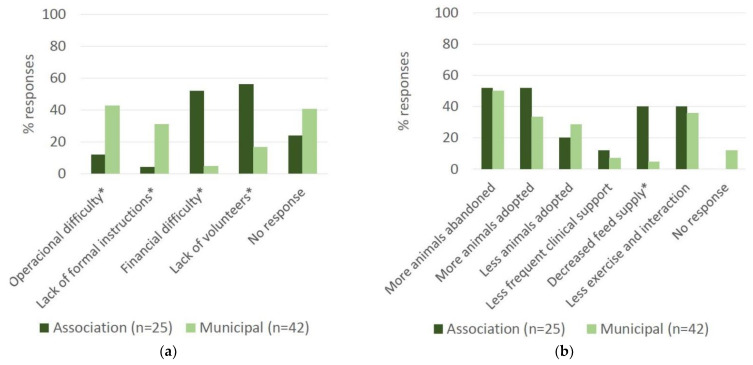
Impact of COVID-19 restrictions on animal shelters: (**a**) impact on routine shelter functioning; (**b**) impact on animal welfare aspects. Data gathered by a questionnaire targeted to Municipal and Non-Municipal shelters (Associations) in March/April 2021. * Indicates statistically significant difference between Municipal shelters and Animal Associations (d.f. 1, *p* < 0.05).

The impact of COVID-19 restrictions on routine shelter functioning is shown in Figure 5a. The first two items were selected by significantly higher proportions of Municipal shelters compared to Associations, whereas the opposite was observed with the two items thereafter. The item “Operational difficulties” was selected by 43% of Municipal shelters and by 12% of Associations (d.f. 1, *p* = 0.009) and “Lack of formal instructions” by 31% of Municipal shelters and by 4% of Associations (d.f. 1, *p* = 0.009). On the contrary, the item “Financial difficulties” was selected by 52% of Associations and by 5% of Municipal shelters (d.f. 1, *p* < 0.001), and “Lack of volunteers” was selected by 56% of Associations and by 17% of Municipal shelters (d.f. 1, *p* < 0.001). A total of 6 (24%) Associations and 17 (40%) Municipal shelters selected the option “No response”.

The impacts of COVID-19 restrictions on certain aspects of animal welfare are shown in Figure 5b. Approximately half of all shelters (52% of Associations; 50% of Municipal shelters) responded that more animals had been abandoned. Adoptions had increased according to 52% of Associations and 33.3% of Municipal shelters, and decreased according to 20% of Animal Associations and 28.6% of Municipal shelters. A decrease in clinical support was reported by 12% of Associations and 7.1% of Municipal shelters. Feed supply was significantly reduced according to 40% of Associations compared to 4.8% of Municipal shelters (d.f. 1, *p* = 0.0003). A total of 40% of Associations and 35.7% of Municipal shelters indicated a reduction in exercise and in the interaction of animals. Except for feed supply, the differences between shelter types were not statistically significant. A total of five (11.9%) Municipal shelters selected the option “No response”. However, one of these did respond “yes” to the item on exercise and interaction.

## 4. Discussion

This study demonstrated differences in the impact of COVID-19 restrictions on Municipal shelters as compared to Associations. The distribution of animals, access to public funding, and the support of functioning by volunteer work were significantly different between the two types of shelter. Considering this, the impacts of COVID-19 restrictions on routine shelter functioning, operational difficulties, the lack of formal instructions, financial restraints and the lack of volunteers were experienced differently by Municipal shelters and Associations. Regarding animal welfare aspects, the decrease in feed supply was also significantly different between shelter types. In the results on abandonment, adoption, exercise and animal–animal/animal–human interactions, and clinical support, there were no statistically significant differences.

The response rate of the questionnaire was similar between Municipal and Association shelters. Overall, responses were representative from all the Portuguese regions of the continent and islands.

The number of dogs and cats was significantly higher in Associations when compared to Municipal shelters. This data may be explained by an increase in social movement towards animal welfare and the necessity of alternatives to the official response to animal abandonment. Currently, in Portugal, as in other countries, Associations play a growing role in the rescue and promotion of the adoption of homeless pets, demonstrating that the responsibility for animal welfare is shared by multiple enactors across society [3,18].

Our study shows that the majority of Associations do not have access to public funding, which may explain the financial difficulties reported by a significantly higher proportion of Associations than by Municipal shelters during the COVID-19 restrictions. Since most Associations depend on private funding and donations to help finance their operations, we speculate that social and economic difficulties caused by the pandemic limited this crucial financial source, as previously reported in the UK [19]. The funding of a shelter has a direct impact on the number of animals that the facility can accommodate. Some of these animals have medical or behavioural issues that need to be addressed before finding suitable homes. All animal shelters must account for these management and medical issues and be able to plan accordingly [20].

According to the questionnaire, Municipal shelters recruited less volunteers than Associations, independently of the pandemic. In Portugal, there is no law addressing volunteer work at Municipal animal shelters. To our knowledge, the main obstacles to volunteer work include biosafety (e.g., transmission of infectious diseases), liability (e.g., insurance) and social concerns (e.g., regular support). Due to the COVID-19 lockdown/restrictions, the animal shelters more affected by the lack of volunteers were Associations, most likely due to the sudden human confinement and inactivity [1]. A decrease in human resources and limited funding may also have influenced feed supply, which was significantly reduced in Associations compared to Municipal shelters. It is essential that Associations develop new strategies to establish current volunteer needs and that they adapt their management programs to future crises or pandemic constraints. They should also be proactive in the dissemination of information regarding the ways volunteers can continue to safely participate in shelter activities.

This study evidenced that the absence of formal instructions and operational difficulties were reported in a significantly higher proportion of Municipal shelters, which are under local governmental (municipal) management, than of Associations, with private management. This is explained by the fact that official organizations have a more formal chain of command and a stricter set of work instructions. Municipal shelters are public health and animal health structures, so staff expected strategic guidelines for action in the context of an emergent pandemic disease. On the other hand, Associations are more dependent on volunteer work, and seem to be more flexible, having a higher adaptability to adverse conditions. Conversely, it is likely that if the pandemic crisis continues to evolve, the overall capacity of the Official Veterinary Services might be compromised [1]. Consequently, it is crucial when facing unpredicted crises to rapidly join efforts of all stakeholders to identify common concerns, exchange information and support, coordinate and develop appropriate responses [3,21,22].

In the present study, more than one third of both shelter types experienced a reduction in exercise and interaction. This is most likely associated with the lack of human resources, staff or volunteers, caused by confinement and eventually medical concerns regarding the transmission and dissemination of the virus between animals and humans [1,9]. Animal welfare outcomes can be achieved by positive human–animal relationships [23]. To increase the physical and psychological well-being of animals kept in confinement, additional volunteers or staff are required to provide exercise and interaction with other animals and humans [11]. This strategy benefits animals beyond their immediate mental health, since it has been demonstrated that regular exercise and handling of shelter dogs has been positively correlated with increased adoption rates [24].

In this survey, nearly half of both shelter types mentioned that more animals had been abandoned during the pandemic restrictions. Even without a pandemic scenario, it is estimated that millions of pets are relinquished and abandoned each year [25]. Based on the available information to date, the risk of animals spreading COVID-19 to humans is considered to be low [26]. Nevertheless, in some countries people have abandoned their animals due to contradictory information on companion animals being potential COVID-19 carriers. Other reasons for abandonment were due to pandemic-related severe health outcomes (e.g., hospitalization), and economic (e.g., unemployment) and social (e.g., moving out of an infected area) concerns [7,9,27]. In other countries, for example, Israel, the levels of abandonment during the pandemic did not change when social isolation was more strictly enforced, due to societal awareness on the human–animal interaction benefits [27].

Although some of the Associations and Municipal shelters reported decreased adoptions during the COVID-19 restrictions, most of them reported an increase, as previously described [10,27]. These differences can be explained by the location of shelters and the population groups closely connected to them. To our best knowledge, adoption was not forbidden at any time period. However, some Municipal shelters were closed to the public between 19 March and 31 May 2020. Conversely, in urban environments, which were affected by the pandemic first, the strict lockdown measures led to a substantial increase in the number of people working from home, and the adoption of animals could have been an incentive to a healthier lifestyle with outside walks [28]. In families and older adults, the benefits of having the routine of taking care of a pet seem to have a positive impact in terms of physical and mental health, providing emotional support [10,29,30]. However, in more fragile social groups affected by physical impairment or economical downfall, animal adoption has not increased, and these factors may have contributed to increased abandonment [7].

A positive aspect evidenced by this national questionnaire was that a reduction in the clinical assistance given to the animals was infrequently reported in all animal shelters. The reported decrease in clinical assistance was slightly higher in Associations than in Municipal shelters. This is possibly due to the fact that the latter work under the responsibility of the municipal veterinarians and their team. Associations can employ one or more veterinarians, and, in some cases, clinical support is provided by external private practices, or private practitioners collaborating as volunteers. During the lockdown, some of these clinical activities may have been temporarily reduced, as reported by other countries [31]. When facing an unforeseen public health event such as the COVID-19 pandemic, in addition to animal disease control, and food safety and security, it is fundamental that governments, Official Veterinary Services and civil society ensure that critical situations are adequately managed when animal welfare is at risk. Preparedness and contingency plans adapted to different types of organizations, such as animal shelters, should be implemented.

One of the limitations of this study is the small sample size of each shelter type. For example, Associations were selected based on a list available from the Official Veterinary Services, so non-registered Associations were not targeted. Regarding the response items of the questionnaire, some were kept short and concise, such as the terms “Operational difficulties” or “Lack of formal instructions”. Future work will focus on the analysis of the remaining questions of the questionnaire to better characterize the two types of animal shelter.

## 5. Conclusions

COVID-19 restrictions had different impacts on the two main types of animal shelters in Portugal. Municipal shelters were more affected by operational difficulties and the lack of formal instructions, and Associations were more vulnerable to external factors since they experienced a decrease in feed supply, volunteers and funding. These differences could be explained by the structural and functional characteristics of each shelter type, such as access to public funding, the number of animals and the number of volunteers. Overall, half of the animal shelters reported increased animal abandonment, as well as increased adoptions. Clinical assistance to the animals was marginally compromised. When facing an unpredicted health and social event such as the pandemic, all sectors of society should rely on preparedness and contingency plans, and these should also be clearly defined for companion animal shelters. Governments, Official Veterinary Authorities, municipalities and non-profit organizations such as Associations should join efforts to ensure animal welfare.

## Data Availability

The data presented in this study is available on request from the corresponding authors.

## Supplementary Materials

The questionnaire is available online at https://www.mdpi.com/article/10.3390/ani11092532/s1. File 1: The questionnaire is available.
