# Supplementary material for: Impact of COVID-19 Restrictions in Portugal: A Questionnaire to Municipal and Animal Association Shelters
Source: Animals (Basel). 2021 Aug 28;11(9):2532. doi: 10.3390/ani11092532 (PMC8472707; doi:10.3390/ani11092532)
Supplement: Supplementary file 1 [file animals-11-02532-s001.zip › animals-1342408-supplementary.pdf]

Secção A: I. INFORMAÇÃO GERAL

A1. É Médico/a Veterinário?

- Sim ☐
- Não ☐

A2. Trabalha em Alojamento/ Abrigo Animais?

- Sim ☐
- Não ☐

A3. Qual o tipo de Alojamento pelo qual é responsável?

- Alojamento para Hospedagem sem Fins Lucrativos - com adoção ☐
- Alojamento para Hospedagem sem Fins Lucrativos - santuário ☐
- Centro de Recolha Oficial Municipal ☐
- Centro de Recolha Oficial Intermunicipal ☐
- Outro ☐

Outro

A4.

De acordo com o mapa supra indique a localização do Abrigo:

As Regiões Autónomas da Madeira e Açores estão contempladas nas respostas apesar de não se encontrarem no mapa.

Fonte da imagem: Wikipédia

- Não responde ☐
- Minho ☐
- Trás-os-Montes e Alto Douro ☐
- Douro Litoral ☐
- Beira Alta ☐
- Beira Litoral ☐
- Beira Baixa ☐
- Extremadura ☐
- Ribatejo ☐
- Alto Alentejo ☐
- Baixo Alentejo ☐
- Algarve ☐
- Madeira ☐
- Açores ☐

A5. Do financiamento anual do Alojamento, qual a percentagem de fundos públicos (estatais)?

- 100% ☐
- >80-99% ☐
- >50-80% ☐
- 20-50% ☐
- <20% ☐

A6. Qual o ano de construção do Alojamento?

- Anterior a 2001 ☐
- 2001-2013 ☐
- Após 2013 ☐

A7. Qual o ano das últimas obras de remodelação do Alojamento?

A8. O Alojamento já está registado pela Direção Geral de Alimentação e Veterinária (DGAV)?

- Sim ☐
- Não ☐
- Outro ☐

Outro

A9. O alojamento está concebido para que animais?

	Sim	Não
Cães	☐	☐
Gatos	☐	☐
Outras espécies	☐	☐

A10. Qual o número atual (aproximadamente) de cães?

A11. Qual o número atual (aproximadamente) de gatos?

A12. Qual o número atual de outras espécies?

A13. Se na questão anterior respondeu "outras espécies", indique quais?

A14. Quais os principais modos de entrada dos cães?

- Abandono nas instalações ☐
- Entrega nas instalações pelo detentor ☐
- Proveniente de CROAs ou associações ☐
- Capturado ☐

C11. **No último ano, na sua opinião, verificou-se um aumento de animais errantes devido a restrições de admissão em alojamentos?**

- Sim ☐
- Não ☐
- Não sei ☐

C12. **Na sua opinião, qual o impacto que as seguintes medidas poderão ter no sentido de diminuir a sobrelotação dos Alojamentos?**

	Muito importante	Importante	Pouco importante	Não responde
Sensibilizar para a detenção responsável	☐	☐	☐	☐
Aumentar a lotação e capacidade dos Alojamentos	☐	☐	☐	☐
Permitir a eutanásia para salvaguardar o bem-estar animal	☐	☐	☐	☐
Promover o controlo reprodutivo	☐	☐	☐	☐
Promover a adoção de animais	☐	☐	☐	☐

C13. **Que medidas adicionais sugere para diminuir a sobrelotação dos Alojamentos?**

Secção D: IV. FUNCIONALIDADE

D1. **Recursos Humanos, indique o número atualmente ligado ao Alojamento (inclui temporários):**

	Nenhum	1	2	3	4	5 - 10	>10
Total de colaboradores	☐	☐	☐	☐	☐	☐	☐
Quantos em exclusividade	☐	☐	☐	☐	☐	☐	☐
Médicos Veterinários	☐	☐	☐	☐	☐	☐	☐
Enfermeiros Veterinários	☐	☐	☐	☐	☐	☐	☐
Tratadores/ assistentes veterinários	☐	☐	☐	☐	☐	☐	☐
Voluntários	☐	☐	☐	☐	☐	☐	☐

D2. **Caso aceitem voluntários, indique:**

	Sim	Não	Não aplicável
Existe oferta regular de voluntários	☐	☐	☐
Há regulamentação própria para voluntariado	☐	☐	☐

D3. **Existe um Regulamento interno de funcionamento implementado?**

- Sim ☐
- Não ☐

D4. **Existe um Plano de lavagem, higienização e desinfeção do Alojamento?**

- Sim ☐
- Não ☐

- Separação cães-gatos ☐
- Enriquecimento ambiental ☐
- Formação em BEA ☐
- Nutrição ☐
- Vacinação ☐
- Desparasitação ☐
- Outro ☐

Outro

K3. **Durante o estado de emergência nacional da COVID-19 observou:**

- Dificuldade operacional ☐
- Falta de instruções oficiais/uniformidade nas regras de admissão ☐
- Dificuldade financeira ☐
- Ausência/diminuição de voluntariado ☐
- Sem resposta ☐

K4. **Considera que o confinamento COVID-19 teve as seguintes consequências:**

- Aumento do abandono ☐
- Aumento da adoção ☐
- Redução da adoção ☐
- Redução na frequência do apoio clínico ☐
- Redução no fornecimento de alimentos ☐
- Redução de passeios e interação ☐
- Sem resposta ☐

K5. **Pode partilhar aqui algumas preocupações, sugestões ou comentários:**

K6. **Se estiver disponível para ser contactado para colaborações futuras sobre Medicina Veterinária de Abrigo, indique um endereço email:**

Muito obrigado pela participação.
